# Diaqua­(2,5-di-4-pyridyl-1,3,4-thia­diazole-κ*N* ^2^)bis­(thio­cyanato-κ*N*)nickel(II) dihydrate

**DOI:** 10.1107/S1600536808030444

**Published:** 2008-09-27

**Authors:** Ming-Hua Yang

**Affiliations:** a Department of Chemistry, Lishui University, Lishui 323000, People’s Republic of China

## Abstract

In the title mononuclear complex, [Ni(NCS)_2_(C_12_H_8_N_4_S)_2_(H_2_O)_2_]·2H_2_O, the Ni^II^ atom is located on an inversion center and is octa­hedrally coordinated by four N atoms from two 2,5-di-4-pyridyl-1,3,4-thia­diazole (bpt) ligands and two thio­cyanate mol­ecules forming the equatorial plane; the axial positions are occupied by two O atoms of coordinated water mol­ecules. O—H⋯O, O—H⋯N and O—H⋯S hydrogen bonds, involving the uncoordinated water molecules, result in the formation of a sheet structure developing parallel to (021).

## Related literature

For related structures, see: Ma & Yang (2008 ▶); Du *et al.* (2002 ▶); Dong *et al.* (2003 ▶); Gudbjarlson *et al.* (1991 ▶). For related literature, see: Su *et al.* (2005 ▶).
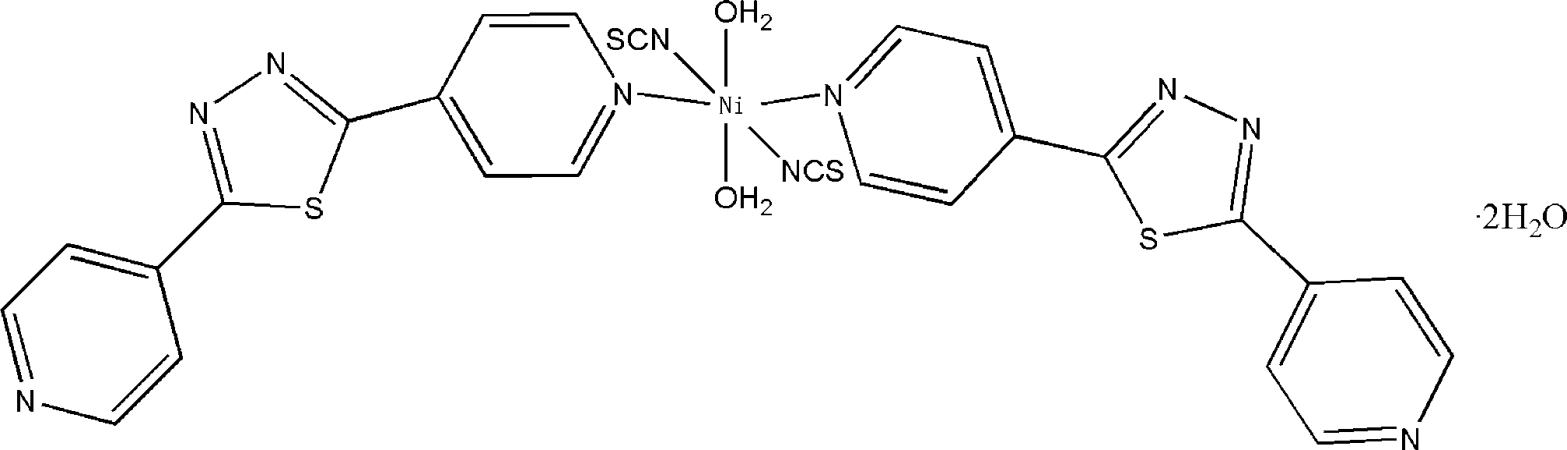


## Experimental

### 

#### Crystal data

[Ni(NCS)_2_(C_12_H_8_N_4_S)_2_(H_2_O)_2_]·2H_2_O
*M* *_r_* = 727.50
Triclinic, 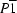

*a* = 7.0555 (11) Å
*b* = 8.3034 (13) Å
*c* = 14.849 (2) Å
α = 104.629 (2)°
β = 93.067 (2)°
γ = 112.228 (2)°
*V* = 768.3 (2) Å^3^
*Z* = 1
Mo *K*α radiation
μ = 0.95 mm^−1^
*T* = 298 (2) K
0.26 × 0.21 × 0.17 mm

#### Data collection

Bruker SMART diffractometer
Absorption correction: multi-scan (*SADABS*; Sheldrick, 1996 ▶) *T*_min_ = 0.789, *T*_max_ = 0.855
3967 measured reflections
2747 independent reflections
1810 reflections with *I* > 2σ(*I*)
*R*_int_ = 0.028

#### Refinement

*R*[*F* ^2^ > 2σ(*F* ^2^)] = 0.055
*wR*(*F* ^2^) = 0.143
*S* = 1.06
2747 reflections
205 parameters
H-atom parameters constrained
Δρ_max_ = 0.39 e Å^−3^
Δρ_min_ = −0.51 e Å^−3^

### 

Data collection: *SMART* (Bruker, 1998 ▶); cell refinement: *SAINT* (Bruker, 1999 ▶); data reduction: *SAINT*; program(s) used to solve structure: *SHELXS97* (Sheldrick, 2008 ▶); program(s) used to refine structure: *SHELXL97* (Sheldrick, 2008 ▶); molecular graphics: *ORTEPIII* (Burnett & Johnson, 1996 ▶), *ORTEP-3 for Windows* (Farrugia, 1997 ▶) and *CAMERON* (Pearce *et al.*, 2000 ▶); software used to prepare material for publication: *SHELXL97*.

## Supplementary Material

Crystal structure: contains datablocks I, global. DOI: 10.1107/S1600536808030444/dn2378sup1.cif

Structure factors: contains datablocks I. DOI: 10.1107/S1600536808030444/dn2378Isup2.hkl

Additional supplementary materials:  crystallographic information; 3D view; checkCIF report

## Figures and Tables

**Table 1:** Hydrogen-bond geometry (Å, °)

*D*—H⋯*A*	*D*—H	H⋯*A*	*D*⋯*A*	*D*—H⋯*A*
O1*W*—H1*WB*⋯O2*W*	0.85	1.91	2.762 (5)	175
O2*W*—H2*WB*⋯N4^i^	0.85	2.00	2.833 (5)	170
O1*W*—H1*WA*⋯S2^ii^	0.85	2.47	3.303 (3)	166
O2*W*—H2*WA*⋯S2^iii^	0.85	2.92	3.540 (4)	132

Symmetry codes: (i) 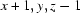; (ii) 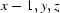; (iii) 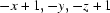.

**Fig. 1.**
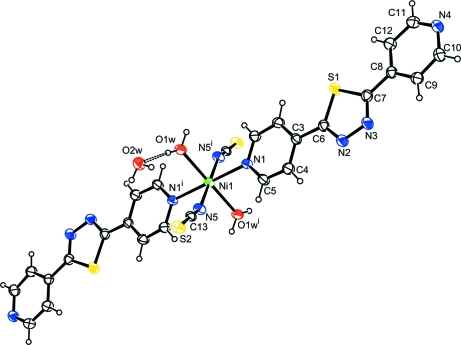
The ORTEP plot of (I), showing the atom-labeling scheme. Displacement ellipsoids are drawn at the 30% probability level. H atoms are represented as small sphere of arbitrary radii. H bond is shown as dashed line. [Symmetry code: (i) 1-x, 1-y, 1-z].

**Fig. 2.**
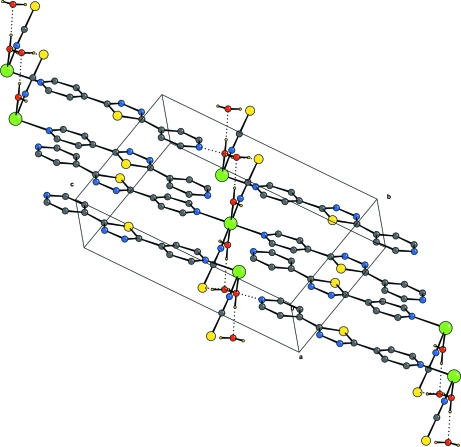
A partial packing view showing the formation of the two dimensional sheet through O-H···O, O-H···N and O-H···S hydrogren bonds. H bonds are represented as dashed lines. H atoms not involved in hydrogen bondings have been omitted for clarity.

## supplementary crystallographic information

### Comment

In the last decades, different kinds of metal-organic frameworks (MOFs) have been synthesized by using linear 4,4'-bipyridine, and other bipyridine-like N,*N*'-donor ligands (Gudbjarlson *et al.*, 1991; Su *et al.* 2005; Dong *et al.*, 2003). However, the angular N,*N*'-ligands were less exploited in building the MOFs in the supramolecular chemistry (Du *et al.*, 2002). In this paper, we report the synthesis and characterization of the title compound (I).

the nickel(II) atom located on an inversion center is octahedrally coordinated by four N atoms from two bpt ligands and two thiocyanate molecules forming the equatorial plane, whereas axial positions are occupied by two O atoms of coordinated water molecules (Fig.1). The Ni—N distances are similar with related complexes (Du *et al.*, 2002; Ma & Yang, 2008).

The occurence of O-H···O, O-H···N and O-H···S results in the formation of a two-dimensional sheet structure developping parallel to the (0 2 1) plane (Table 1, Fig.2). The guest water molecule acts as acceptor and donor.

### Experimental

Bpt (21 mg,0.6 mmol), NiCl2 (28 mg, 0.9 mmol) and NH4SCN (23 mg,0.8 mmol) were added in methanol. The mixture was heated for one hour under refluxing and stirring. The resulting solution was then cooled to room temperature, and some single crystals were obtained five weeks later.

### Refinement

The hydrgen atoms of water molecule were located from difference Fourier maps and their coordinates were initially refined using restraints (O-H= 0.85 (1)Å and H···H = 1.39 (2)Å with *U*iso(H) = 1.5*U*eq(O) then their coordinates were fixed in the last stage of refinement. H atoms attached to C atoms were treated as riding with C-H = 0.93Å and *U*iso(H) = 1.2*U*eq(C).

### Crystal data

**Table:**

[Ni(NCS)_2_(C_12_H_8_N_4_S)_2_(H_2_O)_2_]·2H_2_O	*Z* = 1
*M**_r_* = 727.50	*F*(000) = 374
Triclinic, *P*1	*D*_x_ = 1.572 Mg m^−^^3^
Hall symbol: -P 1	Mo *K*α radiation, λ = 0.71073 Å
*a* = 7.0555 (11) Å	Cell parameters from 2721 reflections
*b* = 8.3034 (13) Å	θ = 1.4–25.2°
*c* = 14.849 (2) Å	µ = 0.96 mm^−^^1^
α = 104.629 (2)°	*T* = 298 K
β = 93.067 (2)°	Block, green
γ = 112.228 (2)°	0.26 × 0.21 × 0.17 mm
*V* = 768.3 (2) Å^3^	

### Data collection

**Table:**

Bruker SMART diffractometer	2747 independent reflections
Radiation source: fine-focus sealed tube	1810 reflections with *I* > 2σ(*I*)
graphite	*R*_int_ = 0.028
φ and ω scans	θ_max_ = 25.3°, θ_min_ = 1.4°
Absorption correction: multi-scan (SADABS; Sheldrick, 1996)	*h* = −8→5
*T*_min_ = 0.789, *T*_max_ = 0.855	*k* = −9→9
3967 measured reflections	*l* = −17→17

### Refinement

**Table:**

Refinement on *F*^2^	Primary atom site location: structure-invariant direct methods
Least-squares matrix: full	Secondary atom site location: difference Fourier map
*R*[*F*^2^ > 2σ(*F*^2^)] = 0.055	Hydrogen site location: inferred from neighbouring sites
*wR*(*F*^2^) = 0.143	H-atom parameters constrained
*S* = 1.06	*w* = 1/[σ^2^(*F*_o_^2^) + (0.0643*P*)^2^ + 0.1149*P*] where *P* = (*F*_o_^2^ + 2*F*_c_^2^)/3
2747 reflections	(Δ/σ)_max_ < 0.001
205 parameters	Δρ_max_ = 0.39 e Å^−^^3^
0 restraints	Δρ_min_ = −0.51 e Å^−^^3^

### Special details

**Table:**

Geometry. All esds (except the esd in the dihedral angle between two l.s. planes) are estimated using the full covariance matrix. The cell esds are taken into account individually in the estimation of esds in distances, angles and torsion angles; correlations between esds in cell parameters are only used when they are defined by crystal symmetry. An approximate (isotropic) treatment of cell esds is used for estimating esds involving l.s. planes.
Refinement. Refinement of *F*^2^ against ALL reflections. The weighted *R*-factor *wR* and goodness of fit *S* are based on *F*^2^, conventional *R*-factors *R* are based on *F*, with *F* set to zero for negative *F*^2^. The threshold expression of *F*^2^ > σ(*F*^2^) is used only for calculating *R*-factors(gt) *etc*. and is not relevant to the choice of reflections for refinement. *R*-factors based on *F*^2^ are statistically about twice as large as those based on *F*, and *R*- factors based on ALL data will be even larger.

### Fractional atomic coordinates and isotropic or equivalent isotropic displacement parameters (Å^2^)

**Table:**

	*x*	*y*	*z*	*U*_iso_*/*U*_eq_	
Ni1	0.5000	0.5000	0.5000	0.0397 (3)	
S1	−0.0367 (2)	0.2796 (2)	0.90422 (9)	0.0507 (4)	
S2	0.7893 (2)	0.05582 (18)	0.46356 (10)	0.0499 (4)	
N1	0.4008 (5)	0.4385 (5)	0.6282 (3)	0.0370 (9)	
N2	0.3264 (6)	0.2926 (6)	0.9413 (3)	0.0507 (11)	
N3	0.2307 (7)	0.2566 (6)	1.0167 (3)	0.0512 (11)	
N4	−0.3536 (7)	0.1274 (6)	1.2103 (3)	0.0530 (11)	
N5	0.6832 (6)	0.3540 (6)	0.4939 (3)	0.0439 (10)	
O1W	0.2492 (4)	0.2685 (4)	0.4104 (2)	0.0456 (8)	
H1WA	0.1300	0.2324	0.4268	0.068*	
H1WB	0.2655	0.1751	0.3792	0.068*	
C1	0.2114 (7)	0.4119 (6)	0.6465 (3)	0.0448 (12)	
H1	0.1217	0.4267	0.6042	0.054*	
C2	0.1394 (7)	0.3639 (7)	0.7239 (3)	0.0479 (13)	
H2	0.0026	0.3409	0.7317	0.058*	
C3	0.2717 (7)	0.3502 (6)	0.7897 (3)	0.0392 (11)	
C4	0.4712 (8)	0.3790 (7)	0.7725 (3)	0.0490 (13)	
H4	0.5656	0.3697	0.8149	0.059*	
C5	0.5267 (7)	0.4217 (7)	0.6915 (3)	0.0454 (12)	
H5	0.6606	0.4399	0.6803	0.054*	
C6	0.2059 (7)	0.3069 (6)	0.8769 (3)	0.0408 (12)	
C7	0.0422 (8)	0.2485 (7)	1.0080 (3)	0.0435 (12)	
C8	−0.0933 (7)	0.2144 (6)	1.0799 (3)	0.0395 (11)	
C9	−0.0247 (8)	0.1812 (7)	1.1595 (3)	0.0514 (13)	
H9	0.1090	0.1867	1.1704	0.062*	
C10	−0.1584 (8)	0.1398 (8)	1.2222 (4)	0.0573 (15)	
H10	−0.1108	0.1191	1.2760	0.069*	
C11	−0.4142 (8)	0.1632 (7)	1.1355 (4)	0.0516 (13)	
H11	−0.5477	0.1593	1.1273	0.062*	
C12	−0.2923 (7)	0.2065 (7)	1.0682 (3)	0.0459 (12)	
H12	−0.3433	0.2298	1.0160	0.055*	
C13	0.7270 (6)	0.2306 (7)	0.4811 (3)	0.0366 (11)	
O2W	0.3283 (5)	−0.0225 (5)	0.3113 (2)	0.0604 (10)	
H2WA	0.3641	−0.0509	0.3583	0.091*	
H2WB	0.4342	0.0242	0.2871	0.091*	

### Atomic displacement parameters (Å^2^)

**Table:**

	*U*^11^	*U*^22^	*U*^33^	*U*^12^	*U*^13^	*U*^23^
Ni1	0.0396 (5)	0.0406 (5)	0.0449 (6)	0.0187 (4)	0.0124 (4)	0.0176 (4)
S1	0.0513 (8)	0.0705 (10)	0.0435 (8)	0.0301 (7)	0.0153 (6)	0.0290 (7)
S2	0.0508 (8)	0.0442 (8)	0.0686 (9)	0.0269 (6)	0.0198 (7)	0.0260 (7)
N1	0.034 (2)	0.039 (2)	0.041 (2)	0.0152 (17)	0.0086 (17)	0.0156 (18)
N2	0.046 (3)	0.065 (3)	0.045 (3)	0.022 (2)	0.013 (2)	0.024 (2)
N3	0.051 (3)	0.066 (3)	0.042 (2)	0.023 (2)	0.015 (2)	0.027 (2)
N4	0.051 (3)	0.065 (3)	0.046 (3)	0.023 (2)	0.017 (2)	0.023 (2)
N5	0.045 (2)	0.045 (2)	0.055 (3)	0.027 (2)	0.0162 (19)	0.022 (2)
O1W	0.0367 (18)	0.045 (2)	0.053 (2)	0.0146 (15)	0.0120 (15)	0.0130 (16)
C1	0.041 (3)	0.054 (3)	0.044 (3)	0.018 (2)	0.008 (2)	0.024 (3)
C2	0.037 (3)	0.058 (3)	0.048 (3)	0.014 (2)	0.010 (2)	0.024 (3)
C3	0.041 (3)	0.036 (3)	0.038 (3)	0.011 (2)	0.012 (2)	0.010 (2)
C4	0.045 (3)	0.066 (4)	0.046 (3)	0.027 (3)	0.010 (2)	0.026 (3)
C5	0.042 (3)	0.057 (3)	0.045 (3)	0.024 (2)	0.018 (2)	0.021 (3)
C6	0.044 (3)	0.039 (3)	0.037 (3)	0.014 (2)	0.009 (2)	0.012 (2)
C7	0.047 (3)	0.046 (3)	0.039 (3)	0.018 (2)	0.007 (2)	0.015 (2)
C8	0.044 (3)	0.038 (3)	0.037 (3)	0.016 (2)	0.008 (2)	0.013 (2)
C9	0.045 (3)	0.067 (4)	0.046 (3)	0.022 (3)	0.008 (2)	0.024 (3)
C10	0.056 (3)	0.076 (4)	0.043 (3)	0.024 (3)	0.013 (3)	0.026 (3)
C11	0.043 (3)	0.055 (3)	0.059 (4)	0.021 (3)	0.011 (3)	0.019 (3)
C12	0.047 (3)	0.053 (3)	0.049 (3)	0.026 (2)	0.009 (2)	0.025 (3)
C13	0.033 (3)	0.046 (3)	0.035 (3)	0.016 (2)	0.012 (2)	0.018 (2)
O2W	0.057 (2)	0.061 (2)	0.054 (2)	0.0141 (18)	0.0183 (17)	0.0132 (19)

### Geometric parameters (Å, °)

**Table:**

Ni1—N5	2.072 (4)	C1—H1	0.9300
Ni1—N5^i^	2.072 (4)	C2—C3	1.371 (6)
Ni1—O1W^i^	2.116 (3)	C2—H2	0.9300
Ni1—O1W	2.116 (3)	C3—C4	1.385 (6)
Ni1—N1	2.176 (4)	C3—C6	1.481 (6)
Ni1—N1^i^	2.176 (4)	C4—C5	1.374 (6)
S1—C7	1.723 (5)	C4—H4	0.9300
S1—C6	1.724 (5)	C5—H5	0.9300
S2—C13	1.635 (5)	C7—C8	1.480 (6)
N1—C1	1.325 (6)	C8—C12	1.380 (6)
N1—C5	1.328 (6)	C8—C9	1.383 (6)
N2—C6	1.304 (6)	C9—C10	1.373 (7)
N2—N3	1.376 (5)	C9—H9	0.9300
N3—C7	1.303 (6)	C10—H10	0.9300
N4—C11	1.310 (6)	C11—C12	1.382 (7)
N4—C10	1.340 (6)	C11—H11	0.9300
N5—C13	1.153 (6)	C12—H12	0.9300
O1W—H1WA	0.8510	O2W—H2WA	0.8456
O1W—H1WB	0.8497	O2W—H2WB	0.8472
C1—C2	1.371 (6)		
			
N5—Ni1—N5^i^	180.000 (2)	C2—C3—C4	117.8 (4)
N5—Ni1—O1W^i^	88.99 (14)	C2—C3—C6	121.6 (4)
N5^i^—Ni1—O1W^i^	91.01 (14)	C4—C3—C6	120.5 (4)
N5—Ni1—O1W	91.01 (14)	C5—C4—C3	118.6 (4)
N5^i^—Ni1—O1W	88.99 (14)	C5—C4—H4	120.7
O1W^i^—Ni1—O1W	180.0	C3—C4—H4	120.7
N5—Ni1—N1	91.17 (14)	N1—C5—C4	124.0 (4)
N5^i^—Ni1—N1	88.83 (14)	N1—C5—H5	118.0
O1W^i^—Ni1—N1	86.52 (12)	C4—C5—H5	118.0
O1W—Ni1—N1	93.48 (13)	N2—C6—C3	123.7 (4)
N5—Ni1—N1^i^	88.83 (14)	N2—C6—S1	113.8 (3)
N5^i^—Ni1—N1^i^	91.17 (14)	C3—C6—S1	122.4 (4)
O1W^i^—Ni1—N1^i^	93.48 (13)	N3—C7—C8	123.5 (4)
O1W—Ni1—N1^i^	86.52 (12)	N3—C7—S1	113.8 (3)
N1—Ni1—N1^i^	180.000 (1)	C8—C7—S1	122.7 (4)
C7—S1—C6	87.2 (2)	C12—C8—C9	118.1 (4)
C1—N1—C5	116.3 (4)	C12—C8—C7	121.8 (4)
C1—N1—Ni1	122.2 (3)	C9—C8—C7	119.9 (4)
C5—N1—Ni1	121.4 (3)	C10—C9—C8	118.5 (5)
C6—N2—N3	112.5 (4)	C10—C9—H9	120.7
C7—N3—N2	112.7 (4)	C8—C9—H9	120.7
C11—N4—C10	116.8 (4)	N4—C10—C9	123.8 (5)
C13—N5—Ni1	159.3 (4)	N4—C10—H10	118.1
Ni1—O1W—H1WA	120.3	C9—C10—H10	118.1
Ni1—O1W—H1WB	121.7	N4—C11—C12	124.1 (5)
H1WA—O1W—H1WB	107.7	N4—C11—H11	117.9
N1—C1—C2	124.1 (4)	C12—C11—H11	117.9
N1—C1—H1	118.0	C8—C12—C11	118.6 (4)
C2—C1—H1	118.0	C8—C12—H12	120.7
C1—C2—C3	119.1 (5)	C11—C12—H12	120.7
C1—C2—H2	120.5	N5—C13—S2	179.7 (4)
C3—C2—H2	120.5	H2WA—O2W—H2WB	109.2

Symmetry codes: (i) −*x*+1, −*y*+1, −*z*+1.

### Hydrogen-bond geometry (Å, °)

**Table:**

*D*—H···*A*	*D*—H	H···*A*	*D*···*A*	*D*—H···*A*
O1W—H1WB···O2W	0.85	1.91	2.762 (5)	175.
O2W—H2WB···N4^ii^	0.85	2.00	2.833 (5)	170.
O1W—H1WA···S2^iii^	0.85	2.47	3.303 (3)	166.
O2W—H2WA···S2^iv^	0.85	2.92	3.540 (4)	132.

Symmetry codes: (ii) *x*+1, *y*, *z*−1; (iii) *x*−1, *y*, *z*; (iv) −*x*+1, −*y*, −*z*+1.
